# Comparison of Subconjunctival Mitomycin C and 5-Fluorouracil Injection for Needle Revision of Early Failed Trabeculectomy Blebs

**DOI:** 10.1155/2016/3762674

**Published:** 2016-02-16

**Authors:** Wei Liu, Jianrong Wang, Miaomiao Zhang, Yuan Tao, Yan Sun

**Affiliations:** The Second People's Hospital of Jinan, No. 148 Jingyi Road, Jinan 250001, China

## Abstract

*Background*. To compare the efficacy of needle revision with 5-fluorouracil (5-FU) and mitomycin C (MMC) on dysfunctional filtration blebs shortly after trabeculectomy.* Methods*. It is a prospective randomized study comparing needle revision augmented with MMC or 5-FU for failed trabeculectomy blebs.* Results*. To date 71 patients (75 eyes) have been enrolled, 40 eyes in the MMC group and 35 in the 5-FU group. 68 patients (72 eyes) have completed 12-month follow-up, 38 eyes in the MMC group and 34 in the 5-FU group. The mean IOP before and that after needle revision in the MMC group were 26.5 ± 4.3 mmHg and 11.3 ± 3.4 mmHg, respectively (*P* < 0.05), and in the 5-FU group were 27.1 ± 3.8 mmHg and 10.9 ± 3.4 mmHg, respectively (*P* < 0.05). At 12-month follow-up, complete success rates were 57.5% for MMC group and 34.3% for 5-FU group (*P* = 0.042; log-rank test) and 75% and 60% (*P* = 0.145; log-rank test), respectively, for the qualified success. Complication rates between the two groups were not statistically different (*P* > 0.05).* Conclusions*. Needle revision and subconjunctival MMC injection were more effective than needling and subconjunctival 5-FU injection for early dysfunctional filtration blebs after trabeculectomies.

## 1. Background

Trabeculectomy is the most common filtration procedure in the surgical treatment of glaucoma. Despite the increasing use of antifibrotic agents to modulate the wound healing response, bleb failure remains a common complication of glaucoma filtration surgery. Failure of the filtration bleb due to subconjunctival scar formation can constitute a significant problem in achieving satisfactory intraocular pressure (IOP) control after trabeculectomy. The rate of bleb failure has been reported to be as high as 10%–20% [1–5].

Needle revision of a failing filtration bleb with antimetabolite injections (either 5-fluorouracil [5-FU] [6–10] or mitomycin C [MMC]) [11–14] has been shown to be a simple and effective way to reestablish aqueous flow and lower IOP. It is difficult to make a literature comparison between 5-FU and MMC needle revisions because few reports have been published [15, 16] and those available mainly studied late bleb failure following trabeculectomy. To the best of our knowledge, this is the first study to directly compare needle revision with 5-FU and with MMC for failed filtration blebs shortly after trabeculectomy.

## 2. Methods

This is a prospective, comparative case series of 75 eyes (71 patients) that underwent needle revision augmented with MMC or 5-FU for failed trabeculectomy blebs between November 2009 and March 2012. The protocol was reviewed and approved by the Institutional Ethics Committee of The Second People's Hospital of Jinan. Each participant provided written informed consent before any study-related examination or procedure was performed and the study adhered to the tenets of the Declaration of Helsinki. Patients were randomly assigned to receive either subconjunctival MMC or 5-FU according to a computer generated randomization list.

**Figure 1:**
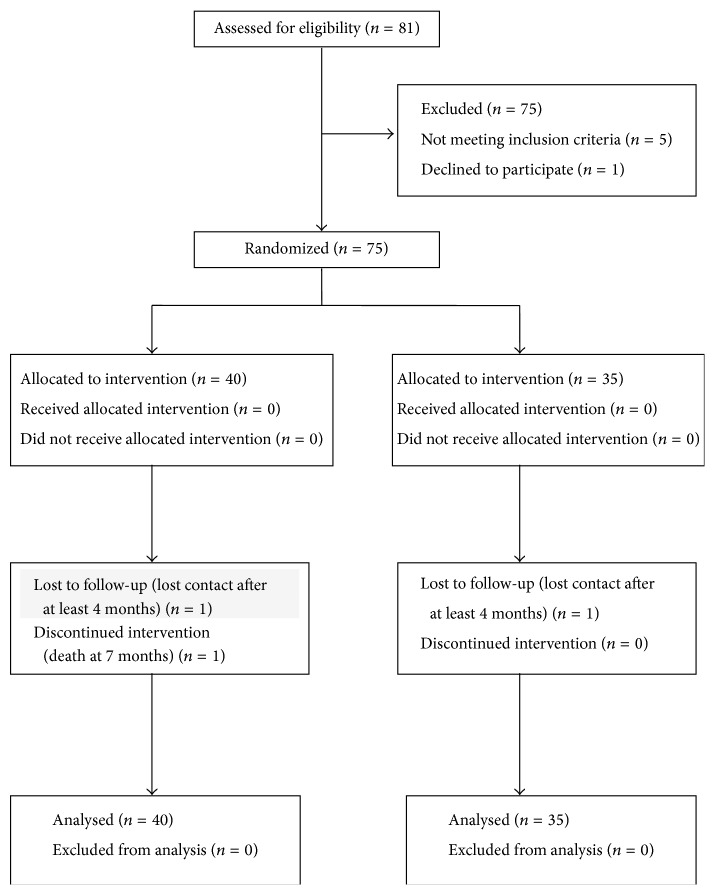
Flow chart for the randomization.

All study eyes had unsuccessful filtering procedures, with or without the use of antifibrotic agents (mitomycin C 0.4 mg/mL was placed under the scleral flap for 1 to 2 minutes before irrigation with balanced salt solution). Bleb massage and suture removal or laser suture lysis were attempted before needle revision. Signs of a failed bleb included an unacceptably high IOP, an open corneoscleral window visible on gonioscopy, vascularization, thickening and flattening of the bleb, and loss of conjunctival microcysts. In all cases gonioscopy was undertaken to ensure that the internal stoma was patent. Patient demographics, glaucoma type, antimetabolite use with trabeculectomy, logMAR best-corrected visual acuity (BCVA), IOP, and time from trabeculectomy were noted. All study eyes had an IOP of >21 mmHg before needle revision. Needling and subconjunctival MMC or 5-FU were applied between 2 and 8 weeks (median = 4.9 weeks) following initial trabeculectomy. Randomization was determined before procedures according to a block randomization sequence prepared by SAS (version 9.1; SAS Institute Inc., Cary, NC, USA). No patients were excluded after the randomization (Figure 1).

A single surgeon (WJR) performed all bleb revisions, using the standard protocol, in an operating room under sterile conditions. A sterile tetracaine-soaked cotton swab was placed over the superotemporal quadrant of the ocular surface for approximately 5 minutes to locally anesthetize the ocular surface. Using a 29-gauge needle, the subconjunctival space was entered at least 10 mm from the filtration bleb site. Subconjunctival fibrosis was disrupted by multiple puncturing motions to restore aqueous drainage. Careful attention was given to avoid inadvertent perforation of the overlying conjunctiva and subconjunctival blood vessels. It was important that the needle insertion under the scleral flap or entering the anterior chamber was avoided. IOP was checked immediately afterward and the needling repeated if IOP had not dropped significantly. At the end of the procedure, a single subconjunctival injection of 5-FU (0.1 mL of 50 mg/mL) or MMC (0.1 mL of 0.2 mg/mL) was administered and the conjunctival sac was rinsed with sterile 0.9% saline. In both procedures, the antiproliferative agent was injected by a separate needle and at least 8 mm away from the bleb limbus, to prevent entry into the anterior.

Patients were examined daily for the first week and at 1, 2, 3, and 6 months and at 1 year. The minimum follow-up was 4 months. During follow-up, BCVA, IOP, glaucoma medications, and complications were recorded at each of the follow-up visits.

Criteria for success were defined before reviewing the data. Complete success was defined as 5 ≤ IOP ≤ 21 mmHg without antiglaucomatous medications measured at the last visit. A qualified success was defined as 5 ≤ IOP ≤ 21 mmHg with topical antiglaucomatous medications. Failure was considered to have occurred from the first visit when the IOP was higher than 21 mmHg and could not be controlled by topical antiglaucomatous medications in eyes. Hypotony was defined as IOP < 5 mmHg. For any patient who was lost to follow-up, success was determined by the clinical status of the patient at the time of the last visit.

Data are presented as mean ± standard deviation. Statistical analyses were performed using SPSS statistical software (ver. 18.0, SPSS, Inc., Chicago, IL). The normality of data was evaluated using an independent sample *t*-test. Descriptive statistics were used to evaluate patient demographic characteristics. Success rates in both groups were compared using Kaplan-Meier life table analysis and the log-rank test. Statistical significance was defined as a *P* value < 0.05.

## 3. Results

Table 1 shows the demographic and clinical characteristics of the patients. No statistically significant differences between the 5-FU and the MMC groups were observed (*P* > 0.05). Baseline IOP before the procedure was 26.5 ± 4.34 mmHg in the MMC group and 27.1 ± 3.85 mmHg in the 5-FU group, a difference that was not statistically significant (*P* = 0.64). Needling and subconjunctival MMC or 5-FU were applied between 2 and 8 (median, 5.3 and 4.5) weeks following trabeculectomy (*P* = 0.054).

Immediately after needle revision, IOP was 11.1 ± 3.4 mmHg (range: 6.0–18 mmHg) in the MMC group and 10.9 ± 3.4 mmHg (range: 6.0–20 mmHg) in the 5-FU group, a slight difference that was not statistically significant (*P* = 0.813). The decrease in IOP following the procedure was statistically significant in both groups (*P* < 0.01).

**Figure 2:**
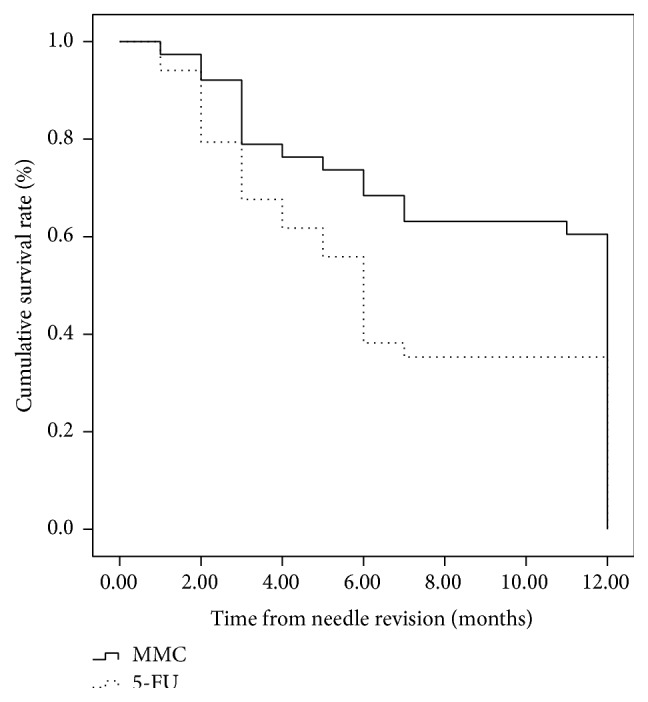
Survival curve with complete success defined as 5 < IOP < 21 mmHg without glaucoma medication (*P* = 0.042).

**Figure 3:**
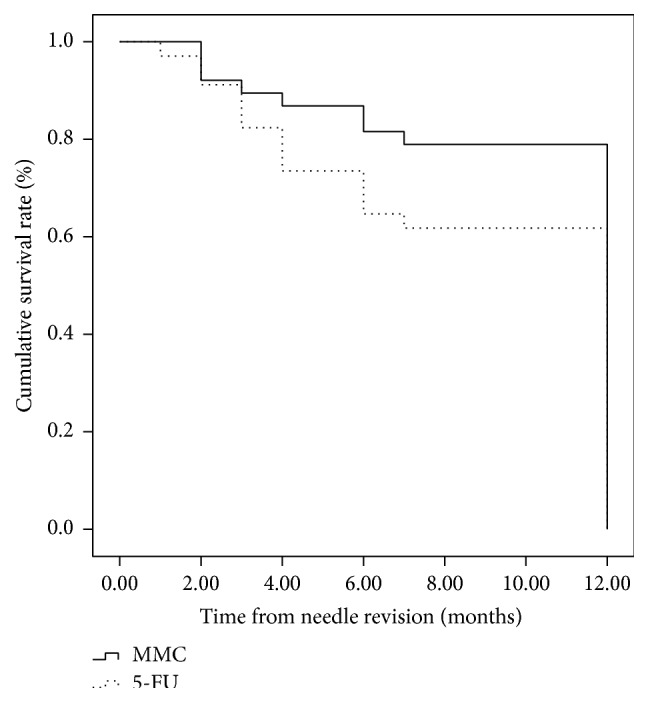
Survival curve with qualified success defined as 5 < IOP < 21 mmHg with and without glaucoma medication (*P* = 0.145).

68 patients (72 eyes) have completed 12-month follow-up, 38 eyes in the MMC group and 34 in the 5-FU group. Twelve-month Kaplan-Meier life table rates for complete success (IOP ≤ 21 mmHg without medications) were 57.5% (23 eyes) and 34.3% (12 eyes) for the MMC group and the 5-FU group, respectively (*P* = 0.042; log-rank test) (Figure 2). The 12-month life table rates for qualified success (IOP ≤ 21 mmHg with medication use) were 75% (30 eyes) and 60% (21 eyes) for the MMC group and the 5-FU group, respectively (*P* = 0.145; log-rank test) (Figure 3).

Complications are listed in Table 2. Complications rates between the two groups were statistically the same. No statistically significant difference between the groups in terms of the occurrence of complications was observed (*P* > 0.05). Leaks through conjunctival entry site persisted for 1 month before resolving in 1 eye of MMC group. Most complications were self-limiting and all complications were resolved without surgical intervention.

## 4. Discussion and Conclusion

Needle revision is considered to be a simple and effective way to rejuvenate failing or failed filtration blebs months, or even years, after trabeculectomy. Since 1990, numerous studies, with variable sample sizes and follow-up periods, have reported various success rates in bleb needle revisions with both subconjunctival 5-FU [3, 6, 8–10, 15, 16] and MMC [11–16]. However, the success rates with both compounds have been highly variable, ranging from 39% to 91% [8, 9, 12, 13, 16] at 12-month follow-up. This is consistent with our findings. In the current study, the total success rates were relatively high, perhaps because revisions were performed sooner after trabeculectomy than other studies. Previous studies mainly examined late bleb failure. Gutiérrez-Ortiz et al. [13] found that the time from the initial filtering surgery to the needling revision was associated with the success rate. Surgery performed less than 4 months previously was found to be a significant factor contributing to the success of the needling procedure. In our study, needle revision was applied between 2 and 8 weeks (median = 4.9 weeks) following initial trabeculectomy. Within 1 month of trabeculectomy, eyes are still early in the wound healing processes. Fibroblast proliferation and early scar formation, which heavily influence filtering bleb morphology and function, are still occurring. Shortly after trabeculectomy, bleb cavities still exist, but the increase in fiber proliferation increases the aqueous flow resistance and can lead to filtering bleb failure. Ren and Qiao [17] proposed that the increased resistance to aqueous flow that results in filtering bleb failure is divided into two parts: those that resist flow upstream from the scleral flap and those that resist flow downstream from the scleral flap. More specifically, shortly after trabeculectomy, there is no resistance to flow upstream to the scleral flap because it is open. However, this is not the case downstream from it because subconjunctival fibrovascular tissue proliferates early in the healing process, creating flow resistance. When bleb needle revision is performed early in this proliferation process, the procedure can have a high success rate. Otherwise, the proliferation moves to the fibers of the scleral surface, causing scleral flap closure and subsequent filtering bleb failure. Eventually, the flap will scar over and, at this time, bleb needle revision is difficult and the procedural success rate is poor.

Palejwala et al. [16] found that there was no apparent difference between the use of 5-FU and the use of MMC. Our results show that subconjunctival MMC is more effective than 5-FU in achieving good pressure control. We believe that there are two reasons. First, wound healing occurs in 3 overlapping phases and in glaucoma filtering surgery, the production, contraction, and remodeling of collagen cause most blebs to fail. A number of factors can inhibit this process, including the use of antiproliferative agents [18]. Considering the 3 phases of wound healing, the most appropriate time to perform MMC needling when signs of failure are detected should be during the cellular phase, which starts several weeks after surgery and continues for months, because MMC inhibits proliferation of fibroblasts during the cellular phase [13]. Second, the biochemical mechanisms of the two drugs are different. Mitomycin C has a greater inhibitory effect on tissues than 5-FU. Because 5-FU is a halogenated pyrimidine analog, it competitively inhibits thymidylate synthetase. The 5-FU becomes incorporated into DNA and RNA within actively replicating cells, leading to defective protein synthesis, and subsequent interference of the cell growth cycle. Therefore, once 5-FU is no longer present, cells that were not in the synthesis phase during drug exposure can still proliferate. On the other hand, MMC is an antibiotic derived from* Streptomyces caespitosus*. It is an alkylating agent, which cross-links DNA, inhibiting mitosis and protein and DNA synthesis. In contrast to 5-FU, it acts at all stages of the cell replication cycle, inhibiting both dividing and resting cells [18]. When applied to the ocular surface topically or injected subconjunctivally, MMC and 5-FU prevent fibroblast proliferation within the subconjunctival space and Tenon's capsule. Mitomycin C also has potent antiangiogenic properties and is thought to have longer-lasting effects than 5-FU on the resident fibroblast population. In animal studies, which compared fibroblast proliferation inhibition with both 5-FU and MMC, the effect of 5-FU only lasted for 7 days, but the effect of MMC was sustained for at least 1 month [19].

Several complications have been reported to be associated with needle revision and subconjunctival [11, 16] MMC or 5-FU application. These include choroidal effusion, shallow anterior chamber, subconjunctival hemorrhage, hypotony maculopathy, and suprachoroidal hemorrhage [16]. In our study, no significant differences in complication rates were observed between the 5-FU and MMC groups. In this study, we had a lower incidence of complications. This is mainly because we only damaged the proliferation of subconjunctival fibers and the needle insertion under the scleral flap or entering the anterior chamber was avoided. The complication that occurred most often in the current study was subconjunctival hemorrhage. This was not a surprise because the needle revision procedure takes place in the subconjunctival space and proliferation of subconjunctival fibers significantly disrupts vascular organization relatively early. All complications that occurred were resolved without surgical intervention.

Our study had limitations. First, in this study, the population were younger and had more angle-closure than the large majority of the needling literature [15, 16], and all patients were of the Chinese race. Young age is associated with needling failure [13]. Mardelli et al. [11] found that successful single-needling procedure was highly correlated with race (white). In future, we can have further study in this regard. Second, the number of complications is small in both groups given the small number of cases in this cohort; thus a significant effect is unlikely to be seen. A prospective, randomized controlled trial with a larger number of cases could resolve these problems.

In conclusion, needle revision and subconjunctival MMC or 5-FU injection was a relatively effective and safe method of treating encapsulated and scarred filtering blebs during the early postoperative stage. Needle revision and subconjunctival MMC injection were more effective than needle revision and subconjunctival 5-FU injection for bleb failure following trabeculectomy.

## Figures and Tables

**Table 1:** Patient demographic and clinical characteristics of the patients.

	MMC group	5-FU group	*P*
Age (year)	49.1 ± 10.4	47.6 ± 10.8	0.542
Gender (F/M)	19/18	23/11	
Baseline IOP (mmHg) [range]	26.5 ± 4.3 [22–38]	27.1 ± 3.8 [23–36]	0.526
BCVA (logMAR)	0.4 ± 0.1	0.4 ± 0.2	1.000
Antimetabolite used in trabeculectomy, number of eyes (%)	34 (85.0)	29 (83.8)	0.801
Time from trabeculectomy to needle revision (week) [range: 2–8 weeks]	5.3 ± 1.90	4.5 ± 1.81	0.066
Diagnosis, number of eyes (%)			
PCAG	23 (57.5)	20 (57.1)	0.385
POAG	14 (35.0)	12 (34.3)	
Juvenile glaucoma	3 (7.5)	1 (2.9)	
Traumatic glaucoma	0 (0)	2 (5.7)	

**Table 2:** Summary of complications that occurred following needle revision.

Complications	MMC group (%)	5-FU group (%)	*P*
Hypotony	3 (7.5)	2 (5.7)	0.756
Corneal punctate epitheliopathy	3 (7.5)	2 (5.7)	0.756
Anterior chamber reaction	5 (12.5)	4 (11.4)	0.887
Subconjunctival hemorrhage	17 (42.5)	14 (40.0)	0.826
Shallow anterior chamber	9 (22.5)	6 (17.1)	0.561
Leak through conjunctival entry site	11 (27.5)	7 (20)	0.446
